# The Protective Effect of the Soluble Egg Antigen of *Schistosoma japonicum* in A Mouse Skin Transplantation Model

**DOI:** 10.3389/fimmu.2022.884006

**Published:** 2022-07-14

**Authors:** Jie Jiang, Junhui Li, Yu Zhang, Chen Zhou, Chen Guo, Zhaoqin Zhou, Yingzi Ming

**Affiliations:** ^1^ Center for Organ Transplantation, Third Xiangya Hospital, Central South University, Changsha, China; ^2^ Research Center of National Health Ministry on Transplantation Medicine Engineering and Technology, Third Xiangya Hospital, Central South University, Changsha, China

**Keywords:** *Schistosoma japonicum*, soluble egg antigen, skin graft, gene expression, cytokine–cytokine receptor interaction

## Abstract

**Background:**

Organ transplantation is currently an effective method for treating organ failure. Long-term use of immunosuppressive drugs has huge side effects, which severely restricts the long-term survival of patients. *Schistosoma* can affect the host’s immune system by synthesizing, secreting, or excreting a variety of immunomodulatory molecules, but its role in transplantation was not well defined. In order to explore whether *Schistosoma*-related products can suppress rejection and induce long-term survival of the transplant, we used soluble egg antigen (SEA) of *Schistosoma japonicum* in mouse skin transplantation models.

**Materials and methods:**

Each mouse was intraperitoneally injected with 100 μg of SEA three times a week for four consecutive weeks before allogenic skin transplant. Skin transplants were performed on day 0 to observe graft survival. Pathological examination of skin grafts was conducted 7 days post transplantation. The skin grafts were subjected to mRNA sequencing. Bioinformatics analysis was conducted and the expression of hub genes was verified by qPCR. Flow cytometry analysis was performed to evaluate the immune status and validate the results from bioinformatic analysis.

**Results:**

The mean survival time (MST) of mouse skin grafts in the SEA-treated group was 11.67 ± 0.69 days, while that of the control group was 8.00 ± 0.36 days. Pathological analysis showed that *Sj* SEA treatment led to reduced inflammatory infiltration within skin grafts 7 days after allogenic skin transplantation. Bioinformatics analysis identified 86 DEGs between the *Sj* SEA treatment group and the control group, including 39 upregulated genes and 47 downregulated genes. Further analysis revealed that *Sj* SEA mediated regulation on cellular response to interferon-γ, activation of IL-17 signaling and chemokine signaling pathways, as well as cytokine–cytokine receptor interaction. Flow cytometry analysis showed that SEA treatment led to higher percentages of CD4^+^IL-4^+^ T cells and CD4^+^Foxp3^+^ T cells and decreased CD4^+^IFN-γ^+^ T cells in skin transplantation.

**Conclusion:**

*Sj* SEA treatment suppressed rejection and prolonged skin graft survival by regulating immune responses. *Sj* SEA treatment might be a potential new therapeutic strategy to facilitate anti-rejection therapy and even to induce tolerance.

## Introduction

Solid organ transplantation is an effective treatment for patients with organ failure. In the past two decades, the rapid development of new immunosuppressive agents and various monoclonal antibody drugs has greatly improved the short-term survival rate of transplants, but the rejection has not been completely overcome, and the long-term survival of transplanted organs has not been significantly improved (1). At the same time, long-term use of immunosuppressive drugs led to high risks of side effects such as drug toxicity, life-threatening infection, and tumors, significantly limiting the long-term survival of patients (2). Thus, there is an urgent need to develop novel immunosuppressant.

Helminths and their secreted products are capable of regulating host immune responses to achieve their ongoing survival, as well as to provide benefit to the host with allergy or autoimmune disorders (3, 4). Recent studies showed that the immunomodulating effect of helminths provided an unintended benefit to the host with allergy or autoimmune disorders, while emerging lines of evidence suggested that helminth-induced immunomodulation has potential in suppressing allograft rejection and promoting transplant tolerance (5, 6). *Schistosoma* is one of the most important parasites in the world, showing high capacity of promoting graft survival in both humans and murine experimental models. In a remarkable clinical study, patients with advanced *Schistosoma mansoni* infection showed significantly prolonged allogenic skin grafts survival when compared with the healthy control group. The mean survival time of allogenic skin grafts in *Schistosoma*-infected patients was 22.25 ± 6.46 days, about 2.21-fold of that in the non-infected group, 10.06 ± 3.21 days (7). Furthermore, no signs of allograft rejection were observed in the remaining 3 patients with schistosomiasis 60 days post allogenic skin transplantation (7). Also, fully allogenic skin transplantation in *S. mansoni*-infected mice demonstrated allograft protection from *Schistosoma* infection. Skin grafts in infected recipients survived 50% longer than those in the control group, when the skin transplantation was performed 60 days post infection (8). Moreover, further analysis indicated a positive correlation between skin graft survival and the burden of live parasites within recipient mice (8). Thus, *Schistosoma* infection exerts a protective role in suppressing rejection and promoting graft survival. *Schistosoma-*derived products represent a potential novel immunosuppressant. However, the active components and the underlying mechanism remain unknown.

*Schistosoma japonicum*, as the main schistosome species in Asia, belongs to the same genus as *S. mansoni*. Although they have a similar capability in regulating host immune responses, the effect of *S. japonicum* infection in transplantation remains to be elucidated. *S. japonicum* eggs and egg-derived products are the main mediators capable of regulating immunological activities; however, its role and the underlying mechanism in transplant immunopathology require further study. In this study, we investigated the therapeutic efficacy of *Sj* soluble egg antigen (SEA) in a fully allogenic skin transplant model in mice. We focused on the immunomodulation effects of *Sj* SEA and explored the related mechanisms *via* bioinformatics analysis and flow cytometry analysis.

## Materials and Methods

### SEA Preparation

Snails infected by *S. japonicum* was provided by the Institute of Schistosomiasis Control, Hunan Province, China. New Zealand white rabbits were infected with cercariae in the snails. After 45 days, the animals were euthanized to obtain and purify mature eggs in the liver (9). Eggs were stored in a concentration of 10^6^ eggs/ml at −80°C. Take 1 ml of worm eggs and add 3–5 ml of PBS to fully grind with liquid nitrogen. After repeated freezing and thawing and homogenization, centrifuge at 4°C, 10,000 × *g* for 60 min. Extract the supernatant SEA and store at a constant concentration of 0.5 μg/μl at −80°C.

### Animal and Experimental Design

Male C57BL/6 and BALB/c mice aged 6–8 weeks were purchased from Hunan Slack Jingda Experimental Animal Company. Mice were housed in the Experimental Animal Center of Central South University, free of specific pathogens, in ventilated cages. The experimental protocol was approved by the Animal Experiment Ethics Committee of Central South University, and all animals received human care according to the principles of laboratory animal care. Before surgery, the recipients (C57BL/6 mice) were pretreated with 100 μg of SEA for 28 days (3 times a week for 4 weeks), and the control group was intraperitoneally injected with PBS. Before skin transplantation, the same batch of C57BL/6 recipient mice and BALB/c donor mice were anesthetized with isoflurane. After the mouse was completely anesthetized, the outer ear (1.0–1.5 cm (2)) of the BALB/c donor mice was cut off with sterile scissors and put into a glassware containing DPBS, and then sterile forceps was used to remove the epidermis and cartilage during ear organization separation. The epidermal layer was placed on a clean gauze and the excess water was dried, and then the epidermal layer was placed with the hair side up and the smooth side down on the transplant bed on the back of the C57BL/6 recipient mice prepared in advance. The transplanted skin should cover the entire transplant bed and there should be no gaps. The skin grafts were fixed with petroleum jelly gauze and sterile bandages, which will be removed for observation after 5 days. The survival of the graft was monitored by daily visual inspection. Rejection was defined as 80% necrosis and dry shrinkage of the grafted skin surface. On the 7th day after the operation, the skin grafts were cryopreserved in liquid nitrogen for genetic analysis. The degree of graft rejection was evaluated on the 9th day after surgery.

### Histology

Skin grafts were removed on the 7th day after transplantation. The tissues were fixed with 10% phosphate buffered formalin, embedded in paraffin, cut into 5-μm sections, and stained with hematoxylin and eosin standard techniques.

### RNA Extraction, Library Construction, and Sequencing

According to the manufacturer’s protocol, use the Trizol kit (Tiangen Biochemical Technology Co., Ltd) to extract total RNA from frozen skin graft tissue. Use Agilent 2100 bioanalyzer (Agilent Technologies, CA, USA) to evaluate the quality and purity of RNA. The library building kit used in the library construction is Illumina NEBNext^®^ UltraTM RNA Library Prep Kit. The mRNA with polyA tail is enriched by Oligo (dT) magnetic beads, and then the resulting mRNA is randomly interrupted with divalent cations in NEB Fragmentation Buffer. Use fragmented mRNA as a template to synthesize cDNA. The purified double-stranded cDNA undergoes end repair, A-tailing, and sequencing. AMPure XP beads (Beckman Coulter, Beverly, USA) are used to screen 200-bp cDNA for PCR amplification and purification of PCR products, and finally a library is obtained. After the library is qualified, the different libraries are pooled according to the effective concentration and target offline data volume and then sequenced by Illumina, and a 150-bp paired-end reading is generated.

### Assemble and Process the Original Data for Sequencing

By removing reads with adapters, removing reads containing N (N means the base information cannot be determined), and removing low-quality reads (Qphred ≤ 20 bases account for the entire read length more than 50% of the reads), we get clean readings. Use HISAT2 v2.0.5 to construct the index of the reference genome, and use HISAT2 v2.0.5 to compare the paired-end clean reads with the reference genome. Use StringTie for new gene prediction (10). FeatureCounts is used to calculate the reads mapped to each gene. Then, calculate the FPKM of each gene based on the length of the gene, and calculate the reads mapped to that gene.

### Differential Expression Analysis

Use DESeq2 R software (1.16.1) to perform differential expression analysis between two comparative combinations (3 biological replicates in each group). DESeq2 provides statistical procedures for determining differential expression in digital gene expression data using models based on the negative binomial distribution. The method of Benjamini and Hochberg was used to adjust the *p*-value to control the false discovery rate. DESeq2 genes with an adjusted *p*-value of <0.05 were assigned as differentially expressed genes (DEGs).

### Enrichment Analysis of DEGs

The GO enrichment analysis of DEGs and the statistical enrichment of DEGs in the Kyoto Encyclopedia of Genes and Genomes (KEGG) pathway were realized by the clusterProfiler R software, which corrects the gene length deviation. Consider that GO terms with corrected *p*-values less than 0.05 are significantly enriched by DEGs. In order to understand the biological functions and pathways of DEGs in the skin grafts of the SEA-treated mice, we used the novemagic cloud platform integrated with annotation and visualization to analyze and visualize the biological functions and pathways of DEGs. Gene set enrichment analysis (GSEA) can be clearly applied to various datasets (11). In this study, GSEA was performed to explore the alteration of potential biological signaling pathways and different biological processes with GSEA software (version 4.1.0). GO and KEGG were used for analysis for the potential altered pathways in SEA-mediated protection on skin graft. We identified the number of random sample permutations as 1,000, and enriched gene sets with a nominal *p* < 0.05 were defined as significant.

### Protein–Protein Interaction Analysis and Network Modules

Protein–protein interaction (PPI) was performed by retrieving a network search tool interacting protein (STRING) database. An interaction score with a median confidence of 0.4 is the standard termination criterion. Subsequently, based on the functional analysis information, the Cytoscape software platform was used to visualize the network. In Cytoscape, cytoHubba was used to identify the hub gene, and Molecular Complex Detection (MCODE) was used for modular analysis. The parameters of cytoHubba used in this study are as follows: the top 15 nodes sorted by degree. The MCODE parameters used in this study are as follows: the degree of cutoff, 2; cluster finding, haircut; node score cutoff, 0.2; k-core, 2; and the maximum depth was 100.

### RT-PCR Analysis

In order to verify the results of RNA-seq analysis, the qPCR method was used to validate the gene expression with SEA-treated and control skin graft samples (*n* = 3), and GAPDH was used as the internal reference. The primer sequence was presented in **Supplementary Table 1**. The 10-μl reaction system included 1 μl of cDNA, 0.1 μl of each primer, 5 μl 2 × SYBR Green qPCR SuperMix (Roche), and 3.8 μl of dH_2_O, and the reaction conditions were as follows: 95°C for 10 min, 94°C for 10 s, 60°C for 20 s, and 60°C for 20 s plate read for 40 cycles followed by melting curve analysis (60°C to 94°C). The 2^−△△Ct^ method was used to determine the relative amount of mRNA, and the measurement was performed at least 3 times independently for each sample.

### Flow cytometry

The following fluorochrome-conjugated antibodies were used for cell surface molecules and flow cytometric analysis: anti-CD4 and anti-CD25. All antibodies were used according to the manufacturer’s instructions. For intracellular staining, spleen cells were restimulated for 4 h with phorbol 12-myristate 13-acetate (50 ng/ml) and ionomycin (550 ng/ml) in the presence of Golgi-Stop. Cells were fixed and made permeable with Cytofix/Cytoperm solution or Foxp3 staining buffer set and were stained with fluorochrome-conjugated anti-Foxp3, anti-IL-4, anti-IL-17, and anti-IFN-γ. All antibodies were used according to the manufacturer’s instructions. All samples were acquired with BD FACS Canto II and data were analyzed with BD FACSDiva software.

### Statistical Analysis

Kaplan–Meier analysis method was used to compare the survival rate of grafts. The results were expressed as mean ± standard deviation. All statistical analysis was performed using GraphPadPrism8 software. The *t*-test was used to determine the differences between 2 groups, and *p* < 0.05 was considered statistically significant.

## Results

### *Sj* SEA Suppresses Allograft Rejection and Prolongs Graft Survival in A Mouse Skin Transplant Model

**Figure 1:**
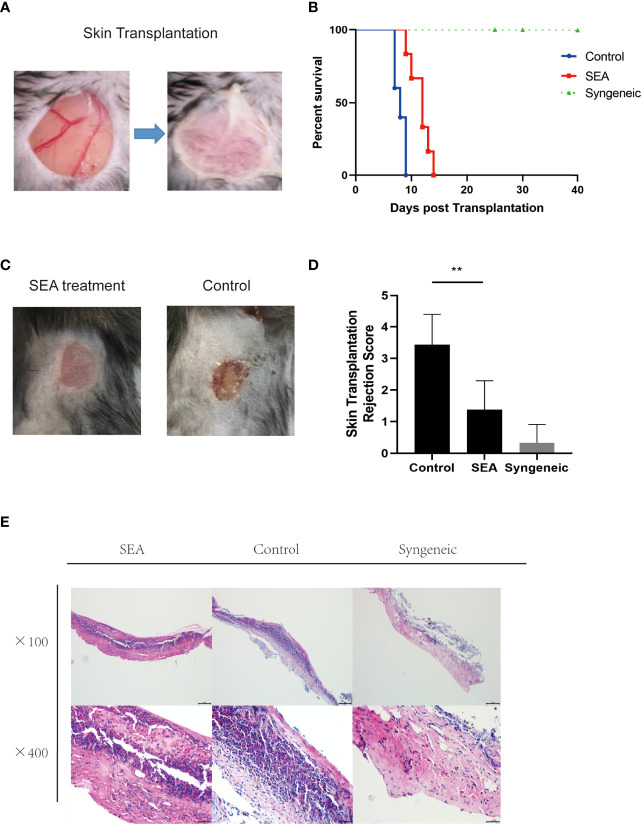
SEA pretreatment can prolong the survival time of mouse skin grafts. **(A)** In the control group (n=5) has no special treatment, and in the treatment group (*n* = 6), each mouse is given SEA 100 μg pretreatment 28 days later (3 times a week, 4 weeks in a row), and the outer ears of BALB/c donor mice were transplanted to the back of C57BL/6 recipient mice. **(B)** Compared with the control group (8.00 ± 0.36), the treatment group (11.67 ± 0.69) significantly prolonged the survival time of skin grafts (*p* < 0.01). **(C, D)** On the 9th day after transplantation, there was no obvious inflammation, ulcer, and necrosis in the skin grafts in the SEA treatment group. Compared with the control group, the graft rejection score was significantly lower than that of the control group (*p* < 0.01). **(E)** The pathological changes of the skin grafts were observed on the 7th day after transplantation. Compared with the control group, the low-power and high-power microscopes showed that the neutrophil cell aggregates decreased.

To explore the effect of SEA in transplantation, we established murine skin transplant models. The treatment group was intraperitoneally injected with 100 μg/mouse SEA 3 times a week for 4 consecutive weeks before transplantation, while the control group was intraperitoneally injected with PBS. Then, we performed allogenic skin transplant (BABL/c to C57BL/6) (**Figure 1A**). Survival analysis showed that the *Sj* SEA treatment significantly prolonged skin graft survival, as the MST of the control group was 8.00 ± 0.36 days while the MST of *Sj* SEA treatment group was 11.67 ± 0.69 days (*p* < 0.01) (**Figure 1B**). Seven days after transplantation, pathological examination was conducted and the graft rejection score was significantly lower in the *Sj* SEA treatment group than that in the control group (*p* < 0.01) (**Figures 1C, D**). Histological analysis of the skin grafts on the 7th day post transplantation revealed that *Sj* SEA treatment resulted in reduced inflammatory infiltration within allograft as compared with the control group (**Figure 1E**).

### Impacts of *Sj* SEA Treatment on the Transcription Profile of Skin Graft

**Figure 2:**
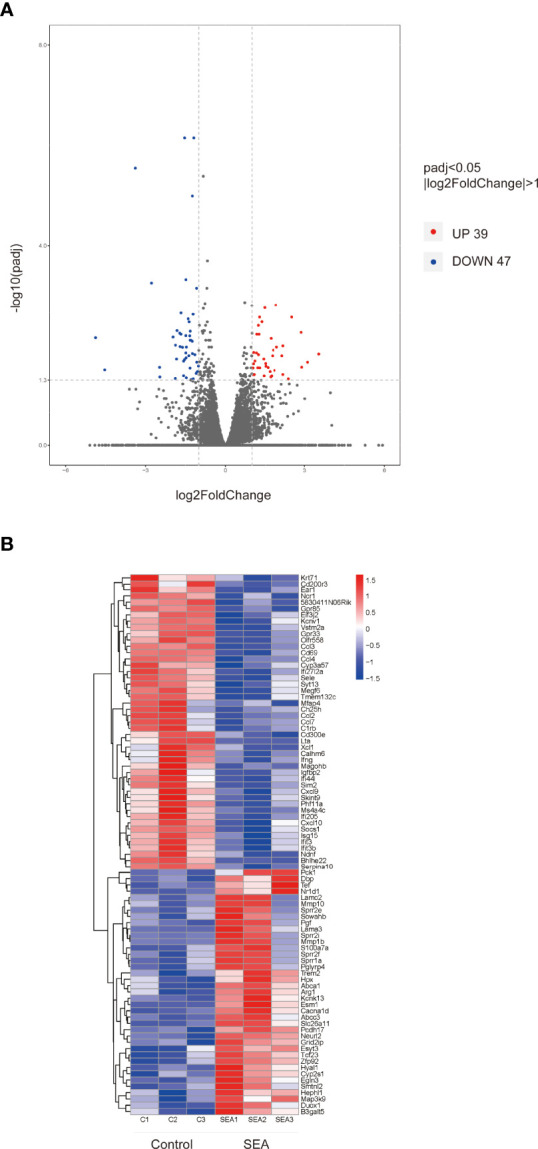
Volcano map and hierarchical clustering heat map of DEGs. **(A)** (|log2Foldchange|>1, *p* adj < 0.05) DEG volcano distribution map; red represents upregulated transcripts; blue represents downregulated transcripts. **(B)** Hierarchical clustering heat map of DEGs (*n* = 3) in each group.

In order to explore the potential molecular mechanism of prolongation of the allogenic graft survival, we performed RNA sequencing analysis on the skin grafts of mice in the SEA group and the control group. Compared with the control group, 86 DEGs were found in the SEA treatment group, including 39 upregulated genes and 47 downregulated genes (|log2Foldchange| > 1, *p* adj < 0.05) (**Figures 2A, B**, (**Supplementary Table 2**). Of note, in these downregulated genes, we found many chemokines such as *Ccl3* and *Ccl4*. The reduction of these chemokines was consistent with reduced inflammatory cell infiltration within graft. Importantly, studies demonstrated that alternatively activated macrophages are correlated transplant protection (12). Among the upregulated genes, *Arg1* was ranked high, which is one of the markers of alternatively activated macrophage.

### GO and KEGG Pathway Analysis of DEGs in *Sj* SEA-Treated Skin Graft

**Figure 3:**
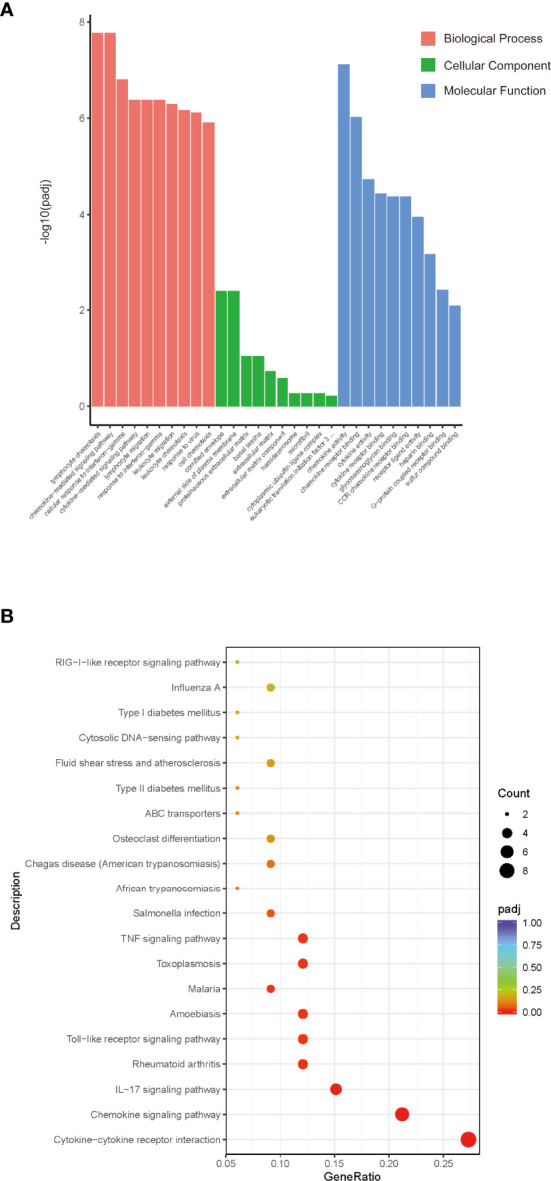
GO and KEGG analysis of the role of DEGs and screening enrichment pathways. **(A)** From the aspects of biological processes, cellular components, and molecular functions, select the top 10 most significant terms and draw a histogram for display. The abscissa in the figure is the description of GO Term, and the ordinate is the significance level of GO Term enrichment. The higher the value, the more significant. Orange represents BP, green represents CC, and blue represents MF. **(B)** Select the 20 most significant KEGG pathways to draw a scatter diagram for display. The abscissa in the figure is the ratio of the number of differential genes annotated to the KEGG pathway to the total number of differential genes, the ordinate is the description of the KEGG pathway, the size of the dot represents the number of genes annotated to the KEGG pathway, and the color from red to purple represents enrichment of the saliency size.

To further investigate the functions of these 86 DEGs and pathways involved in *Sj* SEA-mediated graft protection, we performed GO and KEGG functional enrichment analysis. According to the degree of enrichment of functional annotations, the top 10 biological processes, molecular functions, and cellular components are presented in **Figure 3A** (**Supplementary Table 3**). The gene-annotation enrichment analysis showed that the DEGs in the *Sj* SEA-treated group were related to biological processes including lymphocyte chemotaxis, chemokine-mediated signaling pathway, cellular response to interferon-γ, and cytokine-mediated signaling pathways. Regarding cellular components, the genes changed in the treatment group were mainly related to cornified envelope, external side of plasma membrane, proteinaceous extracellular matrix, and basal lamina (**Figure 3A**, **Supplementary Table 3**). Regarding molecular function, the genes affected by the *Sj* SEA treatment were mainly involved in chemokine activity, chemokine receptor binding, cytokine activity, and cytokine receptor binding (**Figure 3A**, **Supplementary Table 3**). Based on the analysis above, *Sj* SEA-mediated prolongation of graft survival in the skin transplant model may be related to modulation of inflammatory responses within skin grafts.

KEGG pathway analysis was facilitated to understand the possible mechanism of the protective effect of *Sj* SEA in prolonging survival of skin grafts. KEGG results indicated those DEGs involved in more than 20 pathways as shown in **Figure 3B**. Among those related pathways, the cytokine–cytokine receptor interaction ranked first. In this pathway, chemokines such as *Ccl2, Cxcl10*, *Xcl1*, and *Ifng* were downregulated significantly (**Supplementary Table 4**), while some other pathways were associated with inflammation, such as chemokines, IL-17 signaling pathway, Toll-like receptor signaling pathways, as well as TNF signaling pathway.

### GSEA of DEGs in *Sj* SEA-Treated Skin Graft

**Figure 4:**
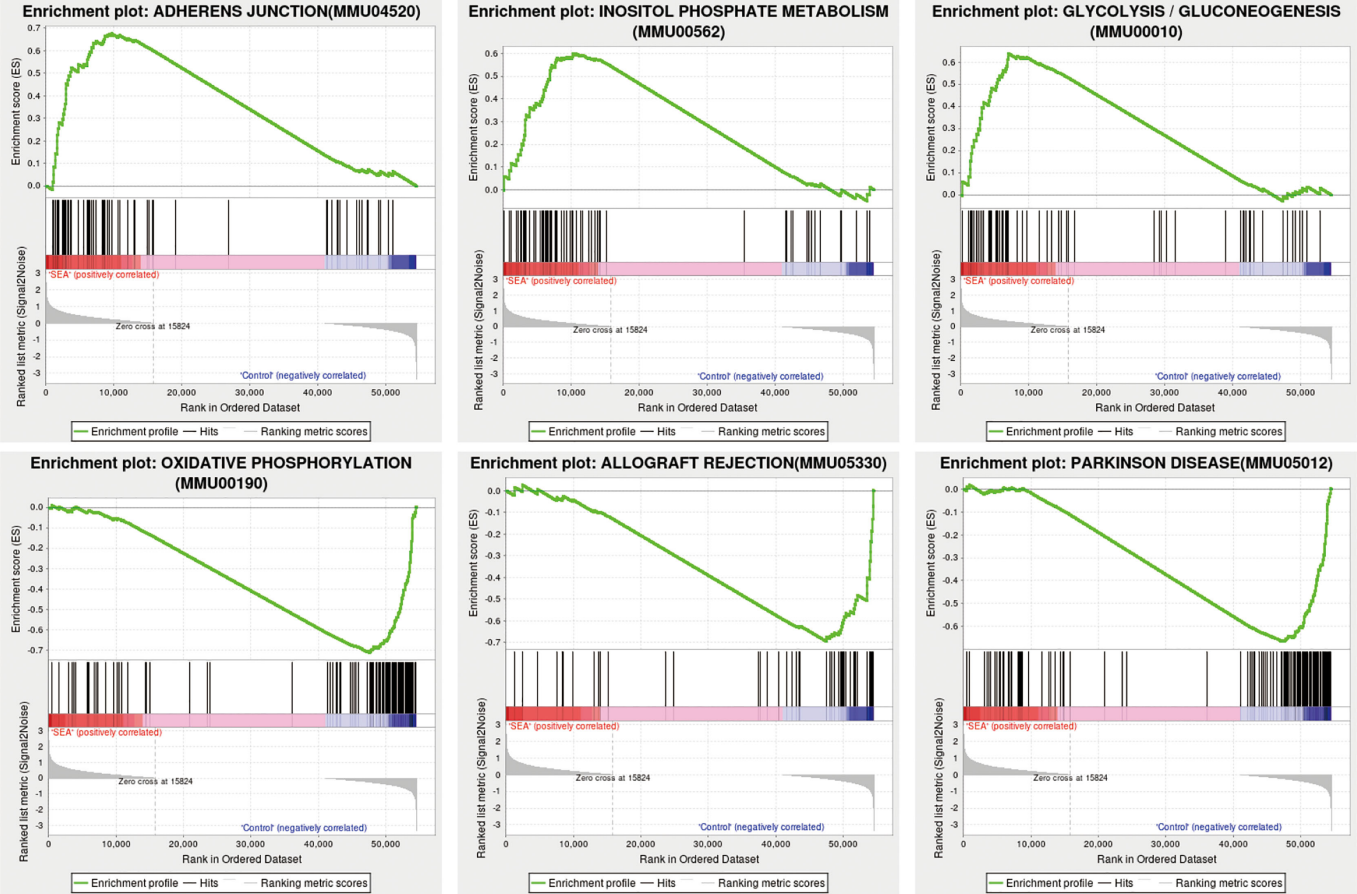
GSEA in *Sj* SEA-treated skin graft. We screened and analyzed 22 upregulated pathways and 22 downregulated pathways based on |NES| > 1 and NOM *p*-value < 0.05. The upregulated enrichment pathways include adherens junction, glycolysis and gluconeogenesis, and inositol phosphate metabolism, among others. The downregulated pathways include oxidative phosphorylation, allograft rejection, Parkinson disease, etc.

To further investigate the possible mechanisms of protection of *Sj* SEA in prolonging the survival of mouse skin grafts, we performed GSEA on the GO and KEGG datasets of this species, respectively. Using the KEGG dataset as the classification standard and screening according to |NES| > 1 and *p* < 0.05, we found that adherens junction, glycolysis and gluconeogenesis, inositol phosphate metabolism, and other pathways were upregulated, while other pathways such as oxidative phosphorylation, allograft rejection, and Parkinson’s disease were downregulated when compared with the control group (**Figure 4**, **Supplementary Tables 5** and **6**). Therefore, GSEA suggested that most of the pathways involved in *Sj* SEA-mediated prolongation of skin graft survival were related to the regulation of energy metabolism, immune-inflammatory response, and oxidative stress.

### Protein–Protein Interaction Analysis Revealed Reduced Expression of Multiple Chemokine Ligands and *Ifng*, and Increased Expression of *Arg1*

**Figure 5:**
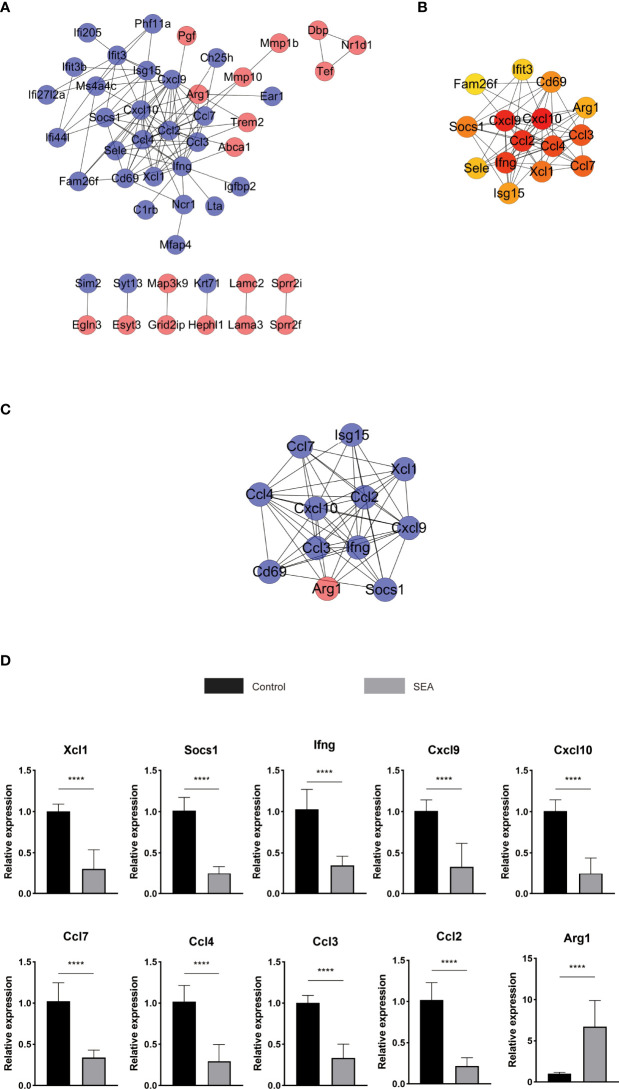
PPI analysis and screening of the hub gene and key signaling pathways in DEGs. **(A)** STRING database analyzes DEGs to get 48 nodes and 106 edges displayed on Cytoscape. Red represents upregulated genes, blue represents downregulated genes, and orange represents hub genes. **(B)** Further analysis with cytoHubba obtained the top 15 most significant genes as hub genes. **(C)** MCODE analysis obtains 12 genes and 52 edges of module 1, MCODE 1 score: 9.455. **(D)** The hub gene of the skin graft obtained by RNA sequencing was verified by qPCR. The significance level was tested by unpaired *t* test (*n* = 3 for each group). The data are shown as the mean ± SEM value. At least 3 independent experiments have also obtained similar results. *p < 0.05; **p < 0.01; ***p < 0.001; ****p < 0.0001.

Using the STRING database to analyze those 86 DEGs, we detected 48 nodes and 106 edges on Cytoscape (**Figure 5A**). Cytoscape and cytoHubba analysis identified 15 hub genes, namely, *Ccl2, Ifng, Cxcl10, Cxcl9, Ccl3, Ccl4, Isg15, CD69, Ccl7, Arg1, Socs1, Ifit3, Xcl1, Sele*, and *Fam26a* (**Figure 5B**). The *Cxcl10* gene had the highest score of 17. Further analysis of those DEGs with the MCODE algorithm showed that MCODE 1 contains 12 gene nodes, similar to the results of the cytoHubba analysis, namely, *Ccl3, Xcl1, Ifng, Ccl2, Cxcl10, Cxcl9, Ccl7, Ccl4, Isg15, Arg1, Socs1*, and *CD69* with 52 edges (**Figure 5C**). Thus, these hub genes might play a critical role in *Sj* SEA-mediated graft protection in murine skin transplant models. Among these hub genes, *Arg1* was an upregulated gene, while others were downregulated genes. These hub genes were mainly involved in cytokine–cytokine receptor interaction and chemokine signaling pathways as indicated by KEGG analysis (**Supplementary Table 4**).

Furthermore, RT-PCR was used to validate the expression of those hub genes involved in *Sj* SEA-induced transplant protection. Compared with the control group, the expression of those identified hub genes, including *Xcl1, Socs1, Ifng, Cxcl9, Cxcl10, Ccl7, Ccl4, Ccl3*, and *Ccl2* were significantly reduced in the *Sj* SEA-treated group, while the expression of *Arg1* was significantly increased (**Figure 5D**). This result was consistent with the results of RNA-Seq analysis.

### Flow Cytometric Analysis of T-Cell Subsets in the Spleen of Mice Before and After Skin Transplantation

**Figure 6:**
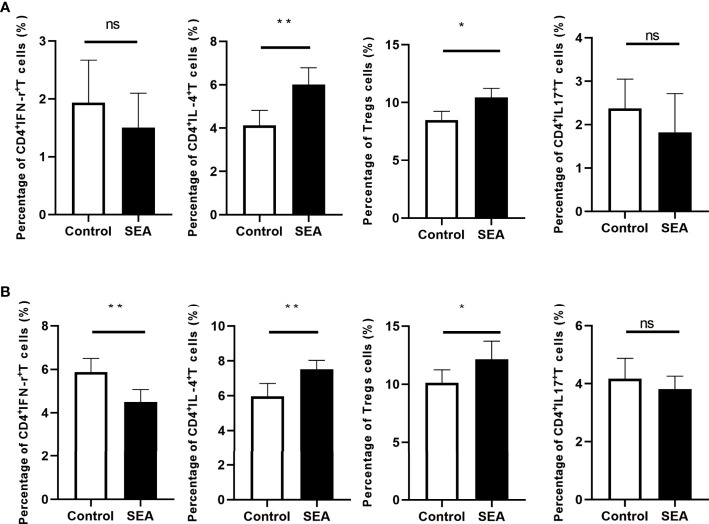
T-cell subsets in the spleen of mice in each group were analyzed by flow cytometry on day 28 after SEA treatment and on day 7 after skin transplantation. **(A)** Compared with the control group, on the 28th day after SEA treatment, the percentages of CD4^+^IL-4^+^ T and Treg cells in the spleen of mice in the experimental group were significantly upregulated, while CD4^+^ IFN-γ^+^ and CD4^+^ IL-17^+^ T-cell ratios were not significantly different. *p  < 0.05, **p  < 0.01, *n* = 6. **(B)** On the 7th day after transplantation, the percentages of CD4^+^ IL-4^+^ T and Treg cells in the spleen of mice in the experimental group were still significantly upregulated compared with the control group, and there was no significant difference in the ratio of CD4^+^ IL-17^+^ T cells, but compared with the control group, the percentage of CD4^+^ IFN-γ^+^ T cells was significantly decreased. *p  < 0.05, **p  < 0.01, *n*  = 6.

The fully allogeneic skin transplant model represents a robust and intense allogeneic reaction, which is mainly mediated by Th1 and Th17 cells (13–15). However, Treg cells play a critical role in suppressing rejection and inducing tolerance (16). Although skin graft transcriptomic data suggested some potential mechanisms of *Sj* SEA-mediated prolongation of skin graft survival, the actual immune cell characterization was needed to be validated. To evaluate the immune status, we performed flow cytometry analysis (gating strategies are shown in **Supplementary Figure 1**). First, 28 days after SEA treatment, before skin transplantation, we used flow cytometry to detect different T-cell subsets. Compared with the control group, we found that the proportion of CD4^+^IFN-γ^+^ T cells in the spleen did not change significantly, while the proportion of CD4^+^IL-4^+^ T cells and CD4^+^Foxp3^+^ Treg cells was upregulated in the SEA treatment group (**Figure 6A**). Subsequently, we sacrificed all mice on the 7th day post transplantation, and continued to use flow cytometry to detect immune status in both groups. Intracellular staining indicated that the proportion of CD4^+^IFN-γ^+^ T cells in the spleen was decreased in the SEA treatment group, while the proportion of CD4^+^IL-4^+^ T cells and CD4^+^Foxp3^+^ Treg cells was both increased. Notably, the proportion of CD4^+^IL-17^+^ T cells in the spleen of the SEA group did not change significantly before and after transplantation when compared with the control group (**Figure 6B**). Thus, flow cytometry analysis indicated that *Sj* SEA-mediated prolongation of skin graft survival was associated with downregulated CD4^+^IFN-γ^+^ T cells and a higher percentage of CD4^+^Foxp3^+^ Treg cells.

## Discussion

In our study, the pretreatment with *Sj* SEA suppressed rejection responses and prolonged murine skin allograft survival by inhibiting allogenic immunopathology. Furthermore, bioinformatics analysis showed that *Sj* SEA-mediated prolongation of skin graft survival was associated with regulation on cellular response to interferon-γ, activation of IL-17 signaling and chemokine signaling pathways, as well as cytokine–cytokine receptor interaction. Flow cytometry analysis demonstrated that prolonged graft survival in the SEA treatment group was associated with higher percentages of CD4^+^IL4^+^ T cells and CD4^+^Foxp3^+^ Treg cells as well as downregulated CD4^+^IFN-γ^+^ T cells. Thus, our results indicated that immunomodulation with *Sj* SEA might have therapeutic potential in transplantation.

It has been found that allogenic skin grafts survived longer in patients with advanced *S. mansoni* infection than those without parasitic infection, highlighting a potential role of chronic *Schistosoma* infection in suppressing rejection (7). Also, allograft protection by *Schistosoma* infection was proved in murine skin transplant models (8). However, this protective effect relied on the duration of infection, as prolonged graft survival was only observed when the transplants were performed 60 days after *S. mansoni* infection, but not 30 days after initial infection. Further analysis indicated that graft survival was positively correlated with worm burden. However, previous studies neglected the impact of schistosome egg, as emerging studies demonstrated the capacity of modulating immune responses by schistosome SEA, particularly in autoimmune disorders and inflammatory diseases (17, 18). Notably, the schistosome egg burden in the recipients was unknown in previous studies; thus, the graft survival difference between 30 days of infection and 60 days of infection may at least partially be the result of different schistosome egg burden. Our data showed that *Sj* SEA pretreatment significantly improved the graft survival. Therefore, *Sj* SEA is capable of inhibiting rejection of skin graft, but the relative contribution remains to be determined. Of note, SEA treatment can avoid the risks of *Schistosoma* infection, which seems to be more practicable in translating into the clinical setting in the future. Although *Sj* SEA, as xenogeneic proteins, will raise the risk of generating neutralizing antibodies and limit the repeated use of *Sj* SEA in the recipients, the underlying mechanism would provide new insights into anti-rejection therapy, and *ex vivo* applications of *Sj* SEA in regulating immune cells seem practicable.

T cells play a key role in transplantation, as T cells are necessary and sufficient to induce rejection. Particularly, Th1 and Th17 cells are critical in determining allograft rejection, as increased Th1 and Th17 cells were observed in rejection while inhibiting Th1 and Th17 cells led to prolonged graft survival (19). Interestingly, *Sj* SEA showed strong capacity to regulate T-cell activities, characterized by impaired Th1/Th17 immune responses, enhanced Th2 immune response, as well as increased Tregs (20–23). In our study, bioinformatics analysis of skin grafts showed that *Sj* SEA treatment led to regulation on cellular response to IFN-γ and activation of IL-17 signaling, thus modulating the effect of Th1 and Th17 cells. Flow cytometry analysis showed that SEA treatment suppressed Th1 cells and upregulated Tregs. Therefore, we inferred that inhibited Th1 and increased Tregs mediated by *Sj* SEA contributed to prolongation of skin graft survival. In addition, we identify 86 DEGs in skin grafts in the SEA-treated group, and for the first time, we identified 10 pathways involved in SEA-mediated skin graft survival prolongation, which may help to understand the mechanisms. Although T cells play critical roles in rejection, other immune cells and non-immune cells are also involved in transplant immunopathology. Several lines of evidence suggested that SEA can also regulate innate immune cells, non-immune cells, metabolism, and apoptosis (23–29). Thus, the protective effects mediated by SEA seems to be multifaceted. However, the active molecules and the underlying mechanisms remain unclear and warrant further study.

Of note, when compared with solid organ transplants, inhibiting skin transplant rejection and inducing tolerance remain a big challenge. The obstacles include high level of resident dendritic cells within skin and colonized microbes mediated Toll-like receptor stimulation. Moreover, Treg-independent mechanisms were also involved in *Schistosoma*-mediated skin graft protection. In our study, we found that *Sj* SEA treatment led to reduced cytokine–cytokine receptor interactions and chemokine signaling pathways. Thus, *Sj* SEA may promote graft survival *via* inhibiting immune cell migration and interaction.

GO analysis showed that among the top 10 most significant pathways in biological processes, cytokine-mediated signaling pathways and other pathways contain many DEGs that express chemokines and related ligands and regulate immuno-inflammatory effects. Interestingly, the hub genes derived from Cytoscape analysis were similar to those derived from MCODE analysis, mainly including *Ccl2, Ccl3, Ccl4, Ccl7, Cxcl9, Cxcl10, Xcl1, Ifng, Arg1*, and *Socs1*. Also, the expression of these genes was validated by RT-PCR. Moreover, most of those genes were concentrated in the cytokine–cytokine receptor interaction pathway, and this pathway was ranked first among the pathways derived from KEGG analysis. These results suggested a potential value of those hub genes in understanding the mechanism.

As mentioned above, KEGG pathway enrichment analysis showed that DEGs are mainly related to cytokine–cytokine receptor interactions and chemokine signaling pathways. These pathways are closely related to the occurrence and development of inflammation and autoimmune diseases. The cytokine–cytokine receptor interaction pathway and the increased expression of *Cxcl9* and *Cxcl10* are related to the development of rheumatoid arthritis (30–33). Moreover, the upregulation of *Ccl2, Ccl3, Ccl4, Ccl7, Cxcl9*, and *Cxcl10* is also related to the acute phase of experimental autoimmune encephalomyelitis (34). The role of these pathways in promoting inflammation and regulating immune response may provide new ideas for SEA in the treatment of other autoimmune diseases and transplant rejection. Although KEGG pathway analysis helped to understand the mechanism to some extent, the significance of this analysis was limited by the amount of DEGs. However, skin graft survival in the *Sj* SEA group was significantly longer than that in the control group, and histological examination also confirmed this phenotype. In addition, it was validated by RT-PCR results and flow cytometry analysis. Thus, the conclusion from KEGG pathway analysis was weak, but it contributed to the mechanism study to some extent.

Among these hub genes, those immunomodulatory genes may play an important role in *Sj* SEA-mediated protection in skin transplantation. Compared with the control group, the expression of chemokines such as *Ccl2, Cxcl9*, and *Cxcl10* was downregulated in the SEA group. These chemokines mainly played a role in the aggregation of neutrophils and the activation of monocytes and lymphocytes (35). The expression of *Arg1* is characteristic on M2 macrophages. Previous studies have found that SEA and its subsets can upregulate the expression of *Arg1* in the host, promoting M2 macrophage polarization and exerting an anti-inflammatory effect (36, 37). IFN-γ is a strong inducer of Th1; however, *Schistosoma* and its secreted products can reduce its expression in the host, reducing the degree of inflammation (38–40). *XCL1* is expressed by various immune cells, including activated CD8 ^+^ T cells, CD4 ^+^ T cells, NK cells, NKT cells, γδ T cells, and thymic medullary epithelial cells (41–46). Similar to *IFN-γ*, the expression of *XCL1* in CD8^+^ T cells, CD4^+^ Th1 cells, and NK cells seems to be related to the Th1-type immune response (42, 44). The decreased expression of *XCL1* further suggested that *Sj* SEA has a regulatory role in host innate immunity and adaptive immunity.

Several lines of evidence have showed that schistosomes and their egg antigens can affect host metabolism (47, 48). A recent study found that *S. japonicum* infection led to changes in the expression of genes related to gut glucose and lipid metabolism, and further investigation identified that SEA changed cellular metabolic responses by inhibiting phosphatase and tensin homolog deleted on chromosome ten (PTEN) in enterocytes (49). In this study, we performed GSEA on the KEGG dataset, and we found that SEA-mediated prolongation of skin graft survival was not only associated with reduced allograft rejection, but also related to alteration in metabolism, such as decreased oxidative stress and increased glycolysis. Although T-cell inflammatory function is associated with increased glycolysis, activated Treg may also use glycolysis for proliferation and when migrating into tissues (50, 51). Flow cytometry analysis showed increased CD4^+^Foxp3^+^ Treg cells and CD4^+^IL4^+^ T cells without a dramatic increase in CD4^+^IFN-γ^+^ T cells and CD4^+^IL17^+^ T cells in the SEA treatment group. Thus, the alteration in metabolism in the skin graft of the SEA treatment group, which was indicated by GSEA, seems to be more correlated with increased CD4^+^Foxp3^+^ Tregs. However, SEA treatment-mediated alteration in metabolism was not specifically determined by both immune cells and non-immune cells, which remains not well defined and requires further study.

In addition to PPI analysis, some upregulated genes also deserve our attention. Nuclear receptor subfamily 1 group D member 1 (*NR1D1*) is a transcriptional repressor that plays an important role in inflammatory responses. *NR1D1* is implicated in the immune system as it has been shown to inhibit Toll-like receptor 4, *Cx3cr1*, and *IL-6* expression in macrophages (52). Also, *NR1D1* regulates the development of Th17 cells and Th17 cell-mediated autoimmune diseases (53). Peptidoglycan recognition proteins (Pglyrps) are a family of innate immune proteins expressed in the skin. Pglyrp4 limits the overactivation of Th17 cells by promoting the accumulation of Treg cells at sites of chronic inflammation, thereby protecting the skin from excessive inflammatory responses to cellular activators and allergens (54). The *CYP2S1* gene is an extrahepatic cytochrome P450 that is constitutively or inducibly expressed in lung, spleen, skin, and some other tissues (55). CYP2S1 may inhibit the expression of many genes in NHEKs, including *IL1β, IL8, IL33, IL36, LL37, CXCL10*, and *CCL20*. These chemokines or cytokines are key mediators of the development of psoriasis (56). *CCL20* was most altered in CYP2S1-overexpressed or CYP2S1-silenced cells, and it was the only receptor for *CCR6*. Multiple studies have shown that the *CCL20/CCR6* axis plays a critical role in the recruitment of Th17 cells to the epidermis and is involved in maintaining the IL23/Th17 signaling pathway (57, 58). *MMP10* is a member of the matrix metalloproteinase (*MMP*) family. Studies have shown that *Mmp10* drives macrophage polarization in the M2 direction, and this mechanism may be related to tolerance through TLR7 signaling (59).

In summary, this study found that *Sj* SEA treatment suppressed rejection and prolonged skin graft survival by regulating immune responses. Bioinformatics analysis identified 86 DEGs between the *Sj* SEA treatment group and the control group, including 39 upregulated genes and 47 downregulated genes. Further analysis indicated that the transplant protection effect was related to *Sj* SEA-mediated regulation on cellular response to interferon-γ, activation of IL-17 signaling and chemokine signaling pathways, as well as cytokine–cytokine receptor interaction. Moreover, *Sj* SEA seemed to modulate both adaptive immunity and innate immunity involved in transplant immunopathology. Therefore, *Sj* SEA treatment might be a new therapeutic strategy to facilitate anti-rejection therapy and even to induce tolerance. However, further study is needed to detect the underlying mechanism.

## Data Availability Statement

The data presented in the study are deposited in the NCBI BioProject repository, accession number: PRJNA844507.

## Ethics Statement

The animal study was reviewed and approved by Institutional Animal Care and Use Committee of Central South University.

## Author Contributions

JJ, JL, YZ, CZ, CG and ZZ performed experiments and analyzed the data. JJ and YZ performed bioinformatic analysis. JJ, JL and YM designed the research and wrote the manuscript. YM supervised the whole project. JL and YM acquired funding for the study. All authors contributed to the article and approved the submitted version.

## Funding

This study was supported by the National Natural Science Foundation of China (81771722, 81901630), Natural Science Foundation of Hunan Province of China (2020JJ5855) and the Key Research and Development Plan of Hunan Province of China (2021SK2032).

## Conflict of Interest

The authors declare that the research was conducted in the absence of any commercial or financial relationships that could be construed as a potential conflict of interest.

## Publisher’s Note

All claims expressed in this article are solely those of the authors and do not necessarily represent those of their affiliated organizations, or those of the publisher, the editors and the reviewers. Any product that may be evaluated in this article, or claim that may be made by its manufacturer, is not guaranteed or endorsed by the publisher.
