# Supplementary figures and images for: The Protective Effect of the Soluble Egg Antigen of Schistosoma japonicum in A Mouse Skin Transplantation Model
Source: Front Immunol. 2022 Jul 14;13:884006. doi: 10.3389/fimmu.2022.884006 (PMC9332893; doi:10.3389/fimmu.2022.884006)

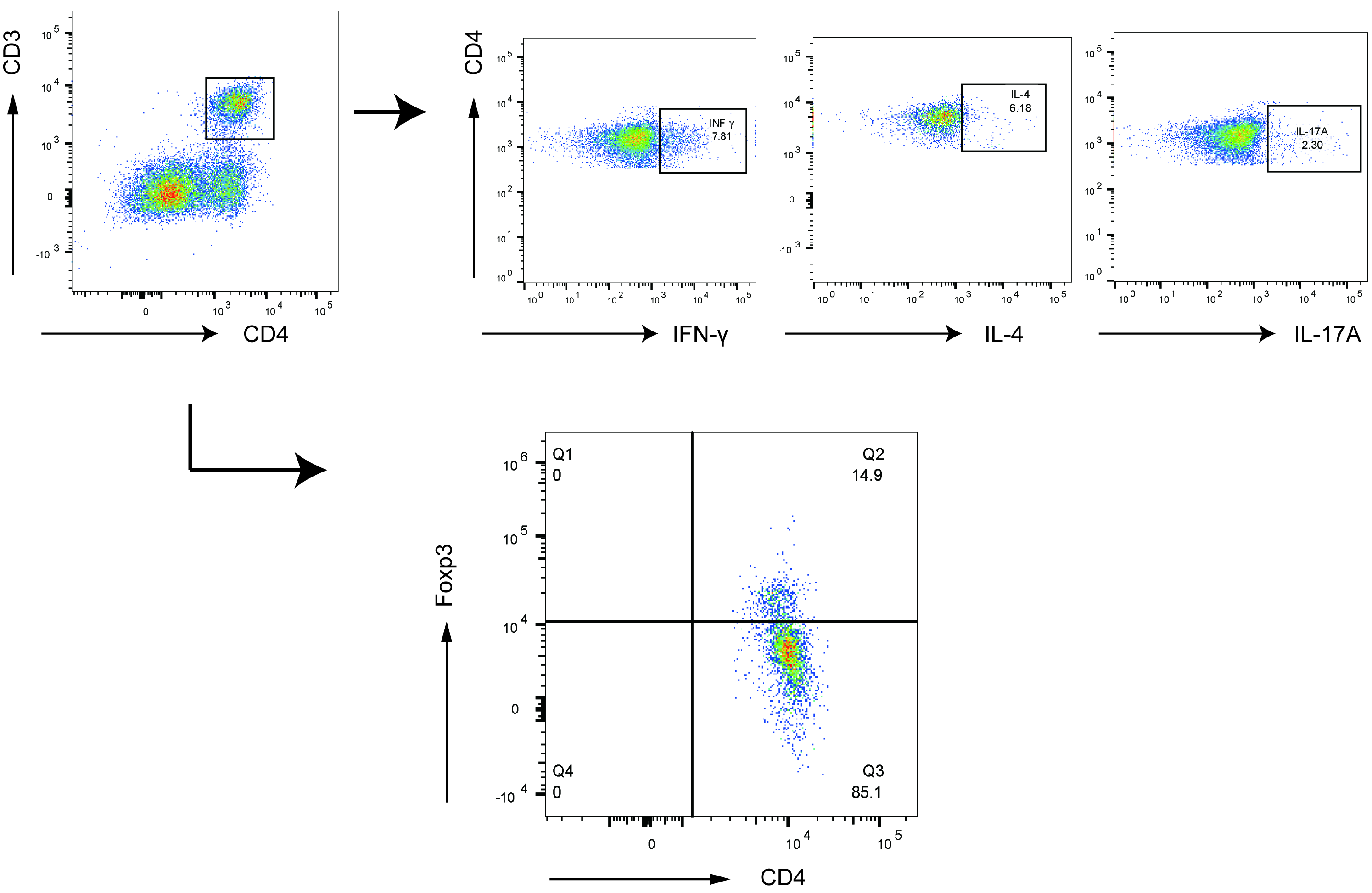

Supplement: Supplementary file 1 [file Image_1.tif]
